# Emendation of Recommendation 6(7), Rule 64 and Appendix 9 Section D of the International Code of Nomenclature of Prokaryotes to regulate the formation of prokaryote names from personal names

**DOI:** 10.1099/ijsem.0.006626

**Published:** 2025-01-03

**Authors:** Aharon Oren

**Affiliations:** 1 Institute of Life Sciences, The Hebrew University of Jerusalem, The Edmond J. Safra Campus, 9190401 Jerusalem, Israel

**Keywords:** diacritic signs, emendation, eponyms, International Code of Nomenclature of Prokaryotes, International Committee on Systematics of Prokaryotes

## Abstract

Following a proposal to emend Recommendation 6(7), Rule 64 and Appendix 9, Section D of the International Code of Nomenclature of Prokaryotes to regulate the formation of prokaryote names from personal names, I hereby report the outcome of the ballot on this proposal by the members of the International Committee on Systematics of Prokaryotes.

## Introduction

In January 2024, M.J.Pallen [1] published a proposal to emend Recommendation 6(7), Rule 64 and Appendix 9, Section D of the International Code of Nomenclature of Prokaryotes (ICNP) [2] to regulate the formation of prokaryote names from personal names. He proposed emendations to the advice currently presented in the ICNP to usher in a modern, consistent, pragmatic and globally inclusive approach to the creation of prokaryotic eponyms.

A public discussion of the proposal was held between 16 January and 15 July 2024 [see Article 13(b) (4) and Article 4(d) of the statutes of the International Committee on Systematics of Prokaryotes (ICSP) [3]]. The collated comments can be found in the attached supplementary information. In response to a comment raised, the author presented an improved version of the proposed rewritten Section D of Appendix 9.

The members of the ICSP voted on the proposals in July–October 2024. E.R.B. Moore (Chair, ICSP) and A. Oren (Executive Secretary, ICSP) collated the results of the vote. Out of the 53 members of the ICSP with voting rights, 30 endorsed the emendations proposed by M.J. Pallen, 7 voted to retain the original versions and 4 abstained. Based on these votes, the following emendations of Recommendation 6(7) and Rule 64 were approved.

### Recommendation 6(7)

The old version (“If genus names or specific epithets are formed from personal names, they should contain only the untruncated family (rarely given) name of a person. Authors should not name organisms after themselves or co-authors.”) is changed to:

**Authors should not name organisms after themselves or co-authors.**

### Rule 64

The old version (“Diacritic signs are not used in the nomenclature of prokaryotes. In names or epithets derived from words with such signs, the signs must be suppressed and the letters transcribed as follows: (1) *ä*, *ö* and *ü* become *ae*, *oe* and *ue;* (2) *é*, *è* and *ê* become *e;* (3) *ø*, *æ* and *å* become *oe*, *ae* and *aa*, respectively.”) is changed to:

**Diacritic signs are not used in the nomenclature of prokaryotes. In names or epithets derived from words with such signs, the signs must be suppressed and the letters transcribed in accordance with established customs for their language of origin.**

The rewritten version of Appendix 9, Section D, as approved, is as follows:

### D. Formation of prokaryote names from personal names

(1) Persons may be honoured by using their name in forming a generic name or a specific epithet. However, the *Code* recommends refraining from naming taxa after persons who are not connected with microbiology or, at least, with natural science (see Recommendations 10 a (1) and 12 c (3)).

(2) *Latinization of personal names* (Table 2).

**Table 2.** Latinization of personal names

**Table:** 

Romanization of the personal name If the personal name originates from a language not written in the roman alphabet, romanize the personal name in accordance with conventions for the language of origin and the preferences of the personal being honoured. When no suitable romanization system exists, transcribe the name phonetically.
Creation of a latinized stem Choose one or more components of the personal name suitable for creationg an agreeable and distinctive scientific name. Avoid components that are too short, too long or otherwise difficult to render in Latin (e.g., containing too few or too many vowels or consonants). Where a single component of the name cannot be on its own used to create an agreeable and distinctive latinized stem, use additional or alternative components of the personal name (e.g. a given name or a given name combined with a family name) or explore additional options for romanization. Remove diacritics, punctuation marks, hyphens and spaces to create a latinized stem. Replacement of characters with diacritics should be undertaken in accordance with customs for the language of origin. If the personal name originates in the German language, characters with umlauts should be replaced by an appropriate digraph: ä is replaced by *ae*, ö by *oe* and ü by *ue*. However, in other languages (e.g., Turkish or Finnish), the vowel with an umlaut should be replaced by the same vowel without the umlaut. For personal names originating from Nordic languages, *ø*, *æ* and *å* become *oe*, *ae* and *aa*. For names of Gaelic origin, expand the patronymic prefix *Mc* to *Mac*. Optional: if the family name ends in a consonant, add *-i* to the original stem to create an augmented stem. Convert the stem to lower case before use in word formation.
Assignment to a declension If the personal name is already a Latin personal name or Latin word, then select the latinized stem and declension already associated with the term (e.g., first declension if the name ends in *-a*, second declension if the name ends in *-us*). The names *Andreas*, *Cosmas*, *Thomas* and *Tobias* are assigned to the first declension with a stem revealed by removing the final as. *Alexander* is assigned to the second declension with the stem *alexandr-*. The names *Michael*, *Raphael*, *Ruben* and *Simon* are assigned to the third declension with stems identical to the nominative form. The names *Felix*, *Leo*, *Paris* and *Vitalis* are assigned to the third declension with stems *felic-*, *leon-*, *parid-* and *vital-*. The name Jesus is treated as an irregular fourth-declension noun with the stem *jesu-* and the genitive form *jesu*. If the personal name ends in *-a*, assign the latinized stem to the first declension. If the personal name ends in *-o*, add *-n* to the end of the name to create a latinized stem and assign the latinized stem to the third declension. Otherwise, assign the stem to the first declension if the name bearer is female or to the second declension if the name bearer is male.

(a) It is a good practice, where possible, to ask the person being honoured by a scientific name for permission to use their name and to consider their preferences for how the name is romanized and latinized.

(b) Authors should refrain from naming bacteria after themselves or co-authors in the same publication (see Recommendations 6(7) and 12c(3)).

(c) The formation of prokaryote names from personal names has no geopolitical meaning and cannot be used to express geopolitical claims (see General Consideration 8).

(d) More than one person can be honoured in a single generic name or epithet. Examples include *Lechevalieria,* after Hubert and Mary Lechevalier and *baumannii,* after Paul and Linda Baumann.

(e) When the personal name is already a Latin name, no further latinization is needed, e.g. Julia, Victoria and Leo. When originating from languages written in non-Roman scripts, personal names should be rendered in the Roman alphabet according to an existing romanization scheme for the name-bearer’s language or, where no such scheme exists, through transcription of the spoken form.

(f) Hyphens and white spaces must be removed from any components of a personal name used to build a scientific name (see Rule 12a). Diacritics must be removed according to the conventions of the language of the name-bearer. For example, the German surnames Höffner and Müller become Hoeffner and Mueller, whereas the Turkish surname Özgür becomes Ozgur and the Finnish surname Törönen becomes Toronen [see Rule 64].

(g) A romanized derivative of the personal name is used to create a latinized stem to which suffixes can be added or which can be used in compound word formation. The choice of which components of the personal name are used to create the latinized stem should consider the wishes of the person being honoured, relevant traditions (e.g. respecting cultures where multiple family names are used or none) and the goal of creating agreeable user-friendly scientific names (see Recommendation 6). When someone has multiple family names, just one or two of these can be used to honour them. Examples include *Boudabousia*, honouring Abdellatif Boudabous Chihi; *Streptomyces lunalinharesii,* honouring Luiz Fernando de Toledo Luna Linhares and *Citrobacter murliniae* honouring Alma C. McWhorter-Murlin.

(h) Although a family name is most often used, generic names or specific epithets can also be formed from given names. Examples include *Erwinia,* after Erwin Smith; *Jutongia,* after Wú Jùtōng; *Staphylococcus arlettae,* after Arlette van de Kerckhove and *Nocardia bhagyanarayanae,* honouring Bhagyanarayana Gaddam. Scientific names can also be formed from given names placed before or after family names conjoined without a connecting vowel. Examples include *Elizabethkingia,* honouring Elizabeth King; *Yonghaparkia,* honouring Yong-ha Park and *Gaoshiqia* honouring Gāo Shíqí.

(i) Kinship prefixes (e.g. Ó/ Ní, Mac/Mc, ibn/bin/bint, Abu, ap and Ben) and particles and articles (e.g. al, de, le, van and von) may be omitted or included in the latinized stem. Examples include *Rochalimaea,* after da Rocha-Lima and *Leclercia,* after Le Clerc. However, honorific titles (e.g. Sir, Sri and Sheikh) are not usually included in prokaryote names.

(j) To render the latinized stems easy to pronounce, simplified or truncated forms of personal names may be adopted. Examples include *chauvoei,* after Chauveau; *Simkania,* after Simona Kahane and *jeikeium* after Johnson and Kaye. Additional vowels may be added (e.g. *macginleyi,* after Kenneth John McGinley). Intentional latinizations involving changes in the orthography of personal names must be preserved (see Rule 60).

(k) When the personal name ends in *-a*, the name should be treated as a first-declension Latin noun, where the stem is formed by removing the *-a*. When the personal name is a Latin name that ends in *-us*, the name should be treated as a second-declension Latin noun, where the stem is formed by removing the *-us*. When the personal name ends in *-o*, the name should be treated as a third-declension Latin noun, where the stem is formed by adding -*n* to give a genitive form ending in *-onis*.

(l) When a family name is used, there is the option to augment the stem by adding *-i* to the end of a family name, e.g. as in the epithet *youngii* in *Xanthomonas youngii*, after John Young. However, this is optional, as seen in *Citrobacter youngae*, which honours Viola Young. Stem augmentation is generally avoided when the latinized stem ends in a vowel, e.g. the epithet *voltae* not *voltiae* from Volta; *sakazakii* not *sakazakiii* from Sakazaki and is inappropriate for components other than family names. Some authorities suggest stem augmentation should be avoided for names ending in *-er*, e.g. in the genus name *Buchnera* or species epithet *brenneri*. However, for consistency, the suffix *-ia* can be used when forming genus names in such cases, e.g. *Listeria* or *Burkholderia*.

(3) *Personal names in generic names* (Table 3)

**Table 3.** Formation of generic names from latinized stems derived from personal names

**Table:** 

To generate a simple genus name from a latinized stem: If the stem belongs to the first declension, add *-aea* to the stem. Example: *Rochalimaea* from the stem *rochalima-* built from the personal name da Rocha Lima. If the stem does not belong to the first declension and ends in a consonant, add *-ia* to the stem. Examples: *Escherichia* from the stem *escherich-* built from the personal name Escherich. If the stem does not belong to the first declension and ends in a vowel, add *-a* to the stem. Examples: *Beneckea* from the stem *benecke-*. Convert the first letter of the stem to upper case. Convert the genus name to italics.
To generate a diminutive genus name from a latinized stem: If the stem ends in *-e* add *-lla*. Example: *Brucella* built from the stem *bruce-*. Otherwise, add *-ella*. Example: *Salmonella* from the stem *salmon-*. Convert the first letter of the genus name to uppercase Convert the genus name to italics.
To generate a compound genus name from a latinized stem: If the stem ends in *-i*, combine the stem and a subsequent word component without a connecting vowel. Example: if the theoretical name *Terasakimonas* were to be formed from the stem *terasaki-* Otherwise, combine the stem and a subsequent word component with the connecting vowel *-i*. Example: *Youngimonas*, built from the stem *young-* from the family name Young. Convert the first letter of the genus name to uppercase. Convert the genus name to italics.

There are three suggested ways to form a generic name from the latinized stem of a personal name:

(a) Directly, by adding the feminine ending -*ia* (or -*a* if the latinized stem ends in *e*, *i* or *u,* or *-aea* when the original personal name ends in *-a*).

(b) As a diminutive, usually by adding the feminine ending *-ella* to the stem. When the stem ends in *-e*, the final letter should be omitted when forming diminutives, e.g., in forming *Brucella* from the personal name Bruce. When digraph *-ee* or the vowel *-y* occurs at the end of personal names, this may be latinized as *i* when forming diminutives. Examples: *Cowdria* and *Roseburia* from Cowdry and Rosebury or the species epithet *asburiae* after the family name Fife-Asbury.

(c) By using a stem derived from a personal name as a word element in a compound name. Here the stem is linked, as needed, to the following word element by a connecting vowel. Example: *Youngimonas*, built from the stem *young-* (derived from the family name Young without stem augmentation), the connecting vowel *-i* and N.L. fem. n. *monas*.

(4) *Personal names in specific epithets* (Table 4).

**Table 4.** Formation of specific epithets from latinized stems derived from personal names

**Table:** 

To generate a genitive species epithet If the stem belongs to the first declension, add *-ae* to the stem. Example: *voltae* from the stem *volt-* from the personal name Volta If the stem belongs to the second declension, add *-i* to the stem. Example: *adleri* from the stem *adler-* from the personal name Adler If the stem belongs to the third declension, add *-is* to the stem. Example: *mayonis* from the stem *mayon-* from the personal name Mayo Convert the species epithet to italics.
To generate an adjectival species epithet If the genus name is masculine, add *-anus* to the stem. Example: *migulanus* from the stem *migul-* from the personal name Migula. If the genus name is feminine, add *-ana* to the stem. Example: *olleyana* from the stem *olley-* from the personal name Olley. If the genus name is neuter, add *-anum* to the stem. Example: *lianum* from the stem *li-* from the personal name Li. Convert the species epithet to italics.

Two possibilities exist to form specific epithets from personal names:

(i) Creation of a genitive noun by adding an inflectional ending to the latinized stem, so that an epithet is formed with the meaning of ‘pertaining to the person being honoured’. The inflectional ending is chosen to reflect the sex of the person to be honoured: typically the feminine ending *-ae* is used for a female or the masculine or neuter ending *-i* is used for a male or non-binary individual, except where a male name belongs to first declension, e.g. *voltae* from Volta or a name from a female belongs to the second declension, e.g. *pistorii* to honour a female with the surname *Pistorius* or where a name of any gender has been assigned to the third declension, e.g. *mayonis* after Mark Mayo. Where the personal name resembles a Latin genitive, it may be used unchanged as a species epithet, e.g. *imshenetskii* to honour Aleksandr Imshenetskii.

(ii) Creation of an adjectival form by adding the endings *-anus* (masculine), *-ana* (feminine) or *-anum* (neuter) to the stem according to gender of the genus name.

*Note*: The changes proposed in this version of Section D take effect on 1 January 2025 and are not retroactive. Names validly published before that date that do not comply with this version of Section D may not be considered to be misspelled for that reason.

## Supplementary material

10.1099/ijsem.0.006626 Uncited Supplementary Material 1.

## Abbreviations

ICNP: International Code of Nomenclature of Prokaryotes
ICSP: International Committee on Systematics of Prokaryotes
