# Supplementary material for: Emendation of Recommendation 6(7), Rule 64 and Appendix 9 Section D of the International Code of Nomenclature of Prokaryotes to regulate the formation of prokaryote names from personal names
Source: Int J Syst Evol Microbiol. 2025 Jan 3;75(1):006626. doi: 10.1099/ijsem.0.006626 (PMC12281833; doi:10.1099/ijsem.0.006626)
Supplement: Uncited Supplementary Material 1. [file ijsem-75-06626-s001.pdf]

Collated Slack comments

Aharon Oren – July 7, 2024

The proposal to emend Recommendation 6, Rule 64 and Appendix 9 of the ICNP by Mark Pallen (Formation of prokaryote names from personal names: a review of current practice and a proposal to emend Appendix 9 of the International Code of Nomenclature of Prokaryotes, https://doi.org/10.1099/ijsem.0.006233) was published on January 16. No discussion comments were yet posted on the Slack channel. The discussion forum will remain open until July 15, midnight. Then the proposal will further be dealt with in accordance with Article 13(b)(4) of the ICSP

Mark Pallen – July 7, 2024

Formation of prokaryote names from personal names: a review of current practice and a proposal to emend Appendix 9 of the International Code of Nomenclature of Prokaryotes
The practice of naming elements from the natural world after notable individuals stretches back to ancient times. This practice of creating eponyms—terms derived from personal names—has been carried forward into prokaryotic nomenclature, where the International Code of Nomenclature of Prokaryotes (ICNP) sets guidelines for creating scientific names from personal names. However, these guidelines can be seen as culturally biased, disjointed and, on occasion, misguided. Here, with the goal of modernizing these recommendations to render them more user-friendly, coherent and inclusive, I review current practice in the light of precedents and key linguistic and cultural principles, while questioning the applicability of the first-name/last-name paradigm for many cultural traditions. Procedural challenges include romanization of the personal name (including handling of diacritics), creation of a short and agreeable latinized stem, assignment of the stem to a declension and addition of suffixes or compound word components to create genus names or species epithets, customizing the approach for names and stems that end in a vowel. I review the pros and cons of stem augmentation, which involves addition of an extra 'i' to the original stem. Next, I formulate a coherent workflow, which I incorporate into a Python script to enable computer-based automation of name creation. Rather than following the ICNP in limiting discussion to a few dozen mainly European names, I examine how these principles work out when applied to the tens of thousands of last names under which scientists publish in the PubMed database, focusing on edge cases where conventional approaches fail, particularly very short and very long names. Drawing on these explorations and analyses, I propose emendations to the advice currently presented in the ICNP to usher in a modern, consistent, pragmatic and globally inclusive approach to the creation of prokaryotic eponyms.

Markus Göker – July 15, 2024

The presentation in the article by Pallen (2024) should have used a table to provide a 1:1 comparison between the current section D of Appendix 9 and the proposed one. It is difficult to see, e.g., whether information would get lost compared to 2022 revision of the ICNP. When

preparing the ballot, the Editorial Board of the ICNP should prepare a 1:1 comparison in order to enable the voting members to make an informed decision.The sentence “When the latinized stem ends in -i, the connecting vowel is omitted.” is highly problematic. First, it does not say “should be” but says “is”. This is retroactive and would imply a potentially high number of orthographic corrections. Similar problems occurred in the proposal to omit the connecting vowel from compound names or epithets in certain situations. The ICNP must be consistent. Names like Youngimonas are compound names. If the phrasing “The connecting vowel is dropped when the following word element starts with a vowel. The connecting vowel may be dropped when the preceding word element ends in the same vowel.”, which is ambiguous enough and may require work of the JC, has now been introduced into Appendix 9, then an analogous phrasing should be used here. For this reason, the sentence should be changed to “When the latinized stem ends in the same vowel, the connecting vowel may be omitted.” The version in the article should better not be ratified.The proposal says that “Tables 2, 3, 4 and 5 should be replaced with Table 1 below”. However, the article by Pallen (2024) does not really demonstrate that Tables 2-5 become useless. They are easier to read than the proposed Table 1 (which should be called Table 2 anyway if it is supposed to be placed after Table 1). They directly provide examples without much text surrounding them. So, I suggest keeping Tables 2-5.There are errors in “Examples chauvoei after Chauveau; Simkania after Simona Kahane, jeikeium after Johnson and Kaye) and additional vowels may be added (e.g. macginleyi after Kenneth John McGinley).” This should be “Examples: chauvoei after Chauveau; Simkania after Simona Kahane; jeikeium after Johnson and Kaye. Additional vowels may be added (e.g. macginleyi after Kenneth John McGinley).”A colon should be appended to sentences like “There are three suggested ways to form a generic name from the latinized stem of a personal name” before an enumeration. Within the enumerations, some paragraphs lack a full stop at the end. The Editorial Board of the ICNP should have a close look at such problems when preparing the ballot.

Aharon Oren – July 16, 2024

The six-month open discussion ended now. The author of the proposal now has up to two months to respond if he wishes, before the ballot will be sent to the ICSP members.

Mark Pallen – July 18, 2024

Replied to a thread: The presentation in the article by Pallen (2024) should have used a table to provide a 1:1 comparison between the current section D of Appendix 9 and the proposed one. It is difficult to see, e.g., whether information would get lost compared to 2022 revision of the ICNP. When preparing the ballot, the Editorial Board of the ICNP should prepare a 1:1 comparison in order to enable the voting members to make an informed decision.…

Thank you for these helpful comments. I have drafted a revised version of the proposal for Section D (attached) that corrects typographical errors and adopts typography consistent with ICNP 2023. I have annotated the revised version with comments which make clear how and why it varies from ICNP 2023 (attached). A key point is the emphasis that scientific names are

built from latinized stems rather than directly from personal names.I have split the table in the original proposal into three tables so as better to preserve the structure of the section and similarly have made adjustments to numbering of sections. Please note that simply preserving the original tables is not an option as they contain multiple erroneous or misleading entries.I have added a statement to the end of the section saying proposed changes are not retroactive. In addition the proposal references Rule 60: "Intentional latinizations involving changes in orthography of personal names must be preserved", which should limit retrospective changes. Note that this posting is to inform the voting process rather than provoke fresh discussion as, in formal terms, public discussion has closed on this proposal. My understanding is that Aharon Oren will include the original published proposal and this modified version of Section D in the voting options.

[This modified Section D is copied in the ballot material]
